# ﻿Review of the genus *Laena* Dejean, 1821 (Coleoptera, Tenebrionidae) from Gansu Province, China, with the description of a new species

**DOI:** 10.3897/zookeys.1190.114201

**Published:** 2024-01-24

**Authors:** Zhonghua Wei, Guodong Ren

**Affiliations:** 1 The Key Laboratory of Southwest China Wildlife Resources Conservation of the Ministry of Education, College of Life Sciences, China West Normal University, 637009, Nanchong, Sichuan Province, China China West Normal University Nanchong China; 2 College of Life Sciences, Hebei University, 071002, Baoding, Hebei Province, China Hebei University Baoding China

**Keywords:** COI gene, DNA barcoding, identification key, Laenini, Lagriinae

## Abstract

A new species of the genus *Laena* from Xiaolongshan in Gansu Province, China is described as *Laenahui***sp. nov.** All *Laena* species known to occur in Gansu Province are reviewed, and an identification key is provided. The mitochondrial gene COI to confirm the identity of the new species, which is morphologically most similar and phylogenetically close to *L.fengileana*. The new species can be recognized by features of elytra and tibiae.

## ﻿Introduction

The genus *Laena* Dejean, 1821 belongs to the tribe Laenini, subfamily Lagriinae, family Tenebrionidae, which is widely distributed in Asia, southern Europe, and southern Africa (Bouchard et al. 2021). Adults are found under loose bark, in leaf litter, in crevices of wood, or under stones in steppe, the alpine zone, and even in subdeserts, while larvae and pupae are found in rotten wood (Wei and Ren 2019a) and in soil for xerophylic species.

In China, the species diversity of the genus *Laena* is extraordinarily high. In the last two decades, more than 100 *Laena* species have been described from China (Schawaller 2001, 2008, 2021; Zhao and Ren 2011, 2012a, 2012b; Schawaller and Aston 2017; Wei and Ren 2019b, 2019c, 2023; Wei et al. 2020, 2021; Schawaller and Bellersheim 2023; Wei and Ren 2023).

Southern Gansu is an important part of the Qingling Mountains, which is an important zone of species diversity in China, and four *Laena* species have been recorded from Gansu Province to date (Reitter 1889; Schawaller 2001, 2008). During an investigation into insect diversity in Xiaolongshan, Gansu Province, another undescribed *Laena* species was collected in southern Gansu.

In this study, *Laenahui* sp. nov. is described and illustrated. An identification key to the five *Laena* species from Gansu is also provided; it is based on examined specimens. DNA barcoding has been widely used in species delimitation in insects (Hebert et al. 2004; Hajibabaei et al. 2006; Smith et al. 2007; Liu et al. 2018; Han et al. 2022; Li et al. 2022) since it was initially proposed by Hebert et al. (2003). To clarify the taxonomic status of the new species, the mitochondrial gene COI was sequenced, and a maximum-likelihood phylogenetic tree was constructed to explore the position of *L.hui* sp. nov. in the genus *Laena*.

## ﻿Materials and methods

The examined *Laena* specimens are deposited in the China West Normal University (**CWNU**), the Museum of Hebei University (**MHBU**), and Institute of Zoology, Chinese Academy of Sciences (**IZAS**). The whole genomic DNA was extracted from leg and thorax muscle tissues of *Laena* specimens using the Ezup Column Animal Genomic DNA Purification Kit (Shanghai, China) following the manufacturer’s instructions. The polymerase chain reactions (PCR) were conducted under the conditions as specified by Wei and Ren (2023). The bidirectional sequencing of mitochondrial gene COI was conducted by Sangon Biotech Co. Ltd (Shanghai, China). The new sequences were checked and edited using SeqMan v. 7.1.0 and BioEdit v. 7.1.11. All the sequences were aligned and trimmed using ClustalW and trimAl v. 1.2, respectively. The best-fit model was calculated using Modelfinder based on the Bayesian information criterion. The maximum-likelihood (ML) tree was constructed using IQtree v. 1.6.8 integrated in PhyloSuite v. 1.2.2 (Zhang et al. 2020) and based on default parameters values. The original ML tree was edited and visualized using FigTree v. 1.43 and Photoshop cc 2019. In total, 31 COI sequences of 17 *Laena* species were used for the phylogenetic analyses, including 25 previously known and the six new sequences provided in this study (Table 1). Five species of the genus *Anaedus* Blanchard, 1842, *Hypolaenopsis* Masumoto, 2001, and *Grabulax* Kanda, 2016 were used as outgroups.

**Table 1.** The taxa were used for phylogenetic analysis in this study.

No.	Taxa	Collection information	GenBank no.	References
GS12	*Laenafengileana* Masumoto, 1996	2022-VII-3, China, Gnasu, Li County, Taopingxiang, Nanshan, elev. 2190 m	OR682144	This study
SC21	*Laenaqinlingica* Schawaller, 2001	2023-IX-8, China, Sichuan, Wangcang County, Micangshan, Shuiliandong elev. 1650 m	OR682145	This study
GS31	*Laenabifoveolata* Reitter, 1889	2022-VI-23, China, Gansu, Qingshui County, Shanmenzhen, Dajicun, elev. 1784 m	OR682146	This study
GS32	*Laenabifoveolata* Reitter, 1889	2022-VI-23, China, Gansu, Qingshui County, Shanmenzhen, Dajicun, elev. 1784 m	OR682147	This study
GS4	*Laenahui* sp. nov.	2021-VIII-29, China, Gansu Province, Tianshui City, Dongchazhen, Dongcha forest farm, elev. 1840 m	OR682148	This study
YN01	*Laenayulongica* Schawaller, 2001	2022-V-7, China, Yunnan, Weixi County, Nilidicun, elev. 2300 m	OR682149	This study
n/a	*Laenahaigouica* Schawaller, 2001	2022-VII-23, China, Sichuan, Songpan, Huanglong, Dawan, elev. 2920 m	OR721926	Wei and Ren 2023
n/a	*Laenakangdingica* Schawaller, 2001	2022-VIII-5, China, Sichuan, Yajiang, Waduozhen, elev. 2600 m	OR721927	Wei and Ren 2023
n/a	*Laenabowaica* Schawaller, 2001	2022-VIII-1, China, Sichuan, Danba, Bianerxiang, Erwacao, elev. 2470 m	OR721930	Wei and Ren 2023
n/a	*Laenabowaica* Schawaller, 2001	2022.VIII.1, China, Sichuan, Danba, Bianerxiang, Erwacao, elev. 2470 m	OR721931	Wei and Ren 2023
n/a	*Laenabifoveolata* Reitter, 1889	2022.VIII.26, China, Gansu, Longnan, Taopingxiang Taoping Forestry Farm, elev. 2576 m	OR721932	Wei and Ren 2023
n/a	*Laenabifoveolata* Reitter, 1889	2022.VIII.26, China, Gansu, Longnan, Taopingxiang Taoping Forestry Farm, elev. 2576 m	OR721933	Wei and Ren 2023
n/a	*Laenapuetzi* Schawaller, 2001	2022.VII.31, China, Sichuan, Jinchuan, Dusongxiang, Dusonggou, elev. 2264 m	OR721934	Wei and Ren 2023
n/a	*Laenamaowenica* Schawaller, 2008	2022.VII.20, China, Sichuan, 6 KM Eastern Mao County, elev. 1896 m	OR721935	Wei and Ren 2023
n/a	*Laenamaowenica* Schawaller, 2008	2022.VII.20, China, Sichuan, 6 KM Eastern Mao County, elev. 1896 m	OR721936	Wei and Ren 2023
n/a	*Laenafengileana* Masumoto, 1996	2022.VII.22, China, Sichuan, Songpan, Mounigou, Shangzhai, elev. 3070 m	OR721937	Wei and Ren 2023
n/a	*Laenabecvari* Schawaller, 2001	2022.VIII.7, China, Sichuan, Litang, Junba, elev. 3050 m	OR721938	Wei and Ren 2023
n/a	*Laenabecvari* Schawaller, 2001	2022.VIII.7, China, Sichuan, Litang, Junba, elev. 3050 m	OR721939	Wei and Ren 2023
n/a	*Laenamounigouica* Wei & Ren, 2023	2022.VII.21, Sichuan, Songpan, Mounigou, Tuguanzhai, elev. 2978 m	OR721941	Wei and Ren 2023
n/a	*Laenamounigouica* Wei & Ren, 2023	2022.VII.21, Sichuan, Songpan, Mounigou, Tuguanzhai, elev. 2978 m	OR721942	Wei and Ren 2023
n/a	*Laenashaluica* Schawaller, 2001	2022.VIII.5, China, Sichuan, Yajiang, Waduozhen, Ridui, elev. 3100 m	OR721943	Wei and Ren 2023
n/a	*Laenashaluica* Schawaller, 2001	2022.VIII.5, China, Sichuan, Yajiang, Waduozhen, Ridui, elev. 3100 m	OR721944	Wei and Ren 2023
n/a	*Laenabarkamica* Schawaller, 2008	2022.VII.26, China, Sichuan, Heishui, Yangyong, Hade, elev. 2600 m	OR721945	Wei and Ren 2023
n/a	*Laenabarkamica* Schawaller, 2008	2022.VII.26, China, Sichuan, Heishui, Yangyong, Hade, elev. 2600 m	OR721946	Wei and Ren 2023
n/a	*Laenafengileana* Masumoto, 1996	2022.VII.23, China, Sichuan, Songpan, Huanglongxiang, Dawan, elev. 2920 m	OR721947	Wei and Ren 2023
n/a	*Laenafengileana* Masumoto, 1996	2022.VII.23, China, Sichuan, Songpan, Huanglongxiang, Dawan, elev. 2920 m	OR721948	Wei and Ren 2023
n/a	*Laenayajiangica* Schawaller, 2001	2022.VIII.4, China, Sichuan, Daofu, Xiatuoxiang, Yiwu, elev. 2780 m	OR721949	Wei and Ren 2023
n/a	*Laenayajiangica* Schawaller, 2001	2022.VIII.4, China, Sichuan, Daofu, Xiatuoxiang, Yiwu, elev. 2780 m	OR721950	Wei and Ren 2023
n/a	*Laenadentithoraxa* Wei & Ren, 2023	2022.VIII.6, China, Sichuan, Yajiang Yizhan, elev. 2800 m	OR721951	Wei and Ren 2023
n/a	*Laenapuetzi* Schawaller, 2001	2022.VII.29, China, Sichuan, Barkman, Shaerzong, Dazatou, elev. 2690 m	OR721952	Wei and Ren 2023
n/a	*Laenapuetzi* Schawaller, 2001	2022.VII.29, China, Sichuan, Barkman, Shaerzong, Dazatou, elev. 2690 m	OR721953	Wei and Ren 2023
n/a	*Hypolaenopsisnomurai* (Schawaller, 2001)	2022.VII.23, China, Sichuan, Songpan, Huanglongxiang, Dawan, elev. 2920 m	OR721929	Wei and Ren 2023
n/a	*Hypolaenopsis* sp.	2022.VII.26, China, Sichuan, Heishui, Yangrong, Hade, elev. 2600 m	OR721940	Wei and Ren 2023
n/a	*Hypolaenopsishongyuanica* (Schawaller, 2001)	2022.VII.26, China, Sichuan, Hongyuan, Shuajingsi, elev. 3160 m	OR721928	Wei and Ren 2023
n/a	*Grabulaxdarlingtoni* Kanda, 2016	Colombia, Sierra Nevada de Santa Marta	KU233834	Kanda et al. 2015
n/a	*Anaedusbrunneus* Ziegler, 1844	n/a	MN448231	Direct submission

## ﻿Results

### ﻿Phylogenetic analyses

**Figure 1.**
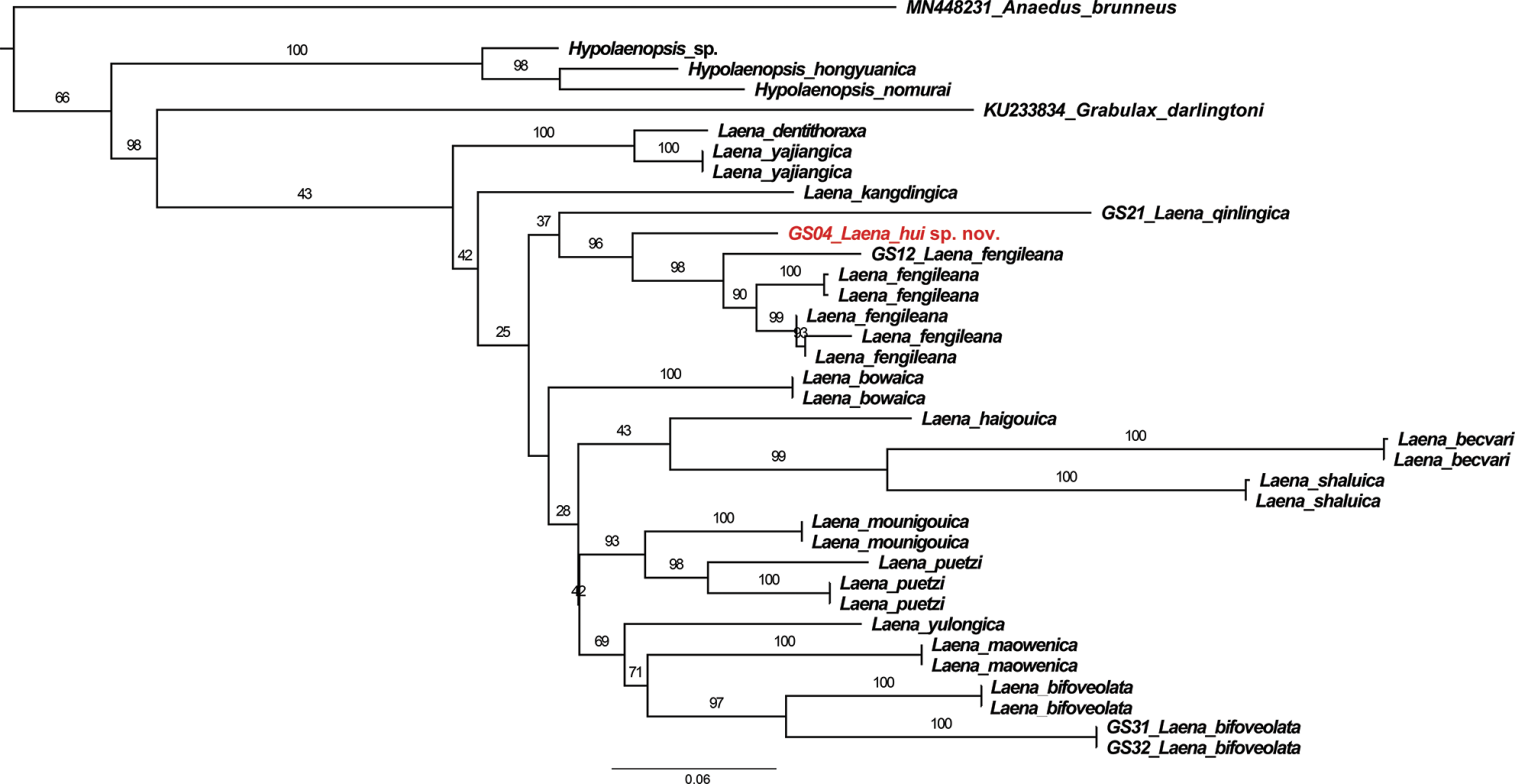
The maximum-likelihood tree of *Laena* species based on mitochondrial gene COI. The new species is in red.

The ML tree was reconstructed based on GTR+I+G4+F model. The phylogenetic tree (Fig. 1) showed that all the *Laena* species form a single clade with low value support (43). The target species, *L.hui* sp. nov., is close to *L.fengileana* in the ML tree with high value support (96).

### ﻿Taxonomy

**Tribe Laenini Seidlitz, 1895**

**Genus *Laena* Dejean, 1821**

### ﻿A key to five *Laena* species recorded from Gansu Province

**Table:** 

1	All femora without teeth on inner sides near apex	**2**
–	All femora with teeth on inner sides near apex	**4**
2	Pronotal lateral margins beaded	**3**
–	Pronotal lateral margins not beaded (Fig. 3A)	***L.bifoveolata***
3	Elytral interval with irregular and small punctures	***L.haigouica***
–	Elytral interval with a row of small punctures	***L.langmusica***
4	Pronotal disc with two impressions in median portion; protibia distinctly broadened at base at inner side; body length 5.7–5.9 mm (Fig. 2H)	***L.fengileana***
–	Pronotal disc without impressions in median portion; protibia gradually broadened from base to apex; body length 7.1–7.7 mm (Fig. 2D)	***L.hui* sp. nov.**

#### ﻿ Laena hui sp. nov.

Taxon classificationAnimaliaColeopteraTenebrionidae

69FF73DE-FCD5-52C9-8BFD-1F2B8B380DFC

https://zoobank.org/DD047226-D95F-4642-BD96-053044595E53

Fig. 2A–G

##### Type locality.

China, Gansu Province, Tianshui City, Dongchazhen.

##### Type specimens.

***Holotype***: China • ♂; Gansu Province, Tianshui City, Dongchazhen, Dongcha forest farm; 34°15'54″N, 106°35'39″E; elev. 1840 m; 2021-8-29; Qi Liu leg.; MHBU. ***Paratype***: China • 1♀ (in 95% ethanol); the same data as holotype; CWNU.

##### Diagnosis.

**Figure 2.**
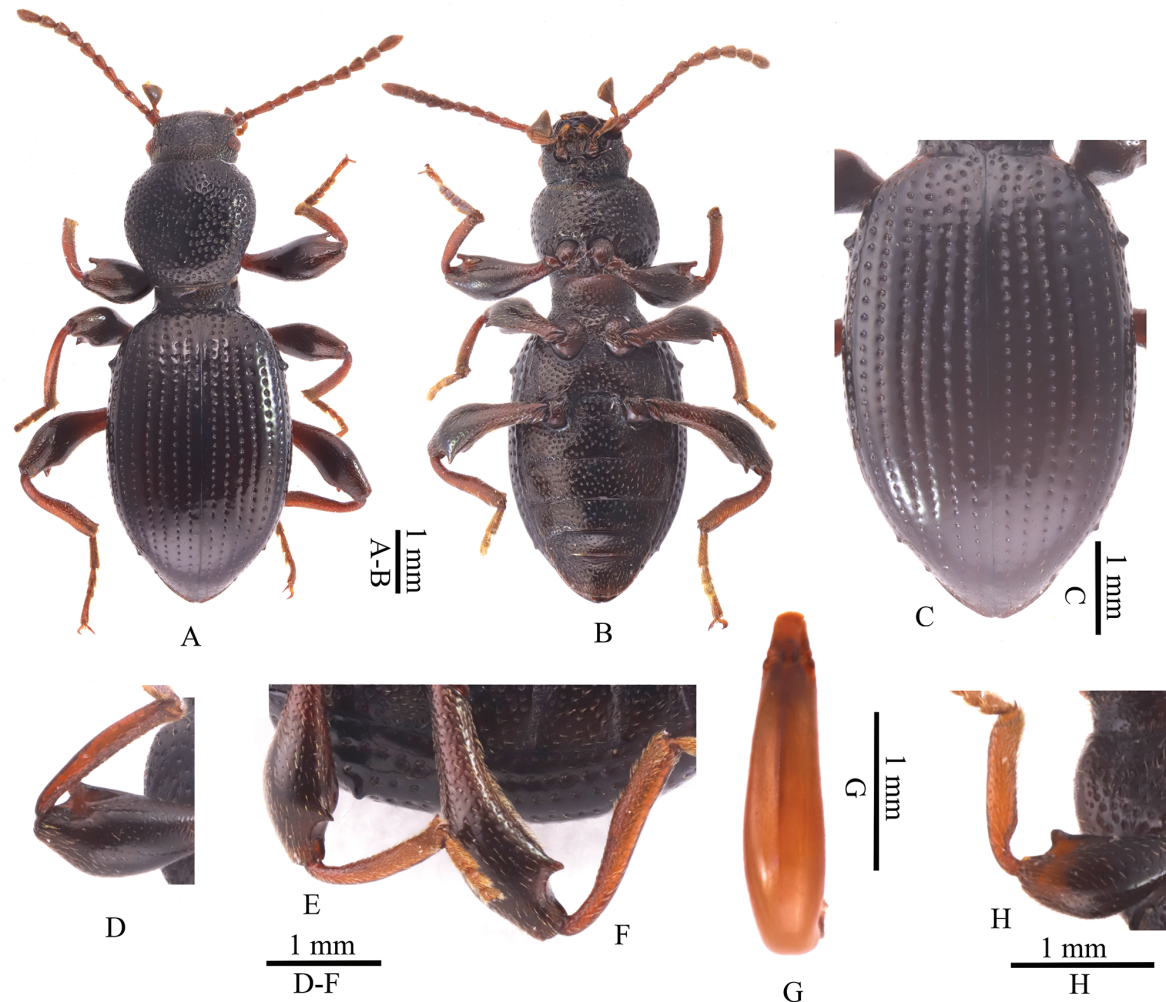
*Laena* species **A–G***Laenahui* sp. nov., holotype **A, B** dorsal and ventral views **C** elytra **D–F** pro- meso- and metaleg, in ventral view **G** aedeagus **H** proleg of *L.fengileana*.

Based on morphological characteristics (as provided in the identification key) and the phylogenetic position in the ML tree, the new species is most similar and closest to *L.fengileana*. However, *L.hui* sp. nov. can be distinguished from *L.fengileana* by the following characters: body larger, length 7.1–7.7 mm (5.7–5.9 mm in *L.fengileana*); body surface with very short setae, elytral intervals glabrous (elytral intervals each with a row of small punctures in *L.fengileana*); all tibiae distinctly hooked at their inner apex, protibiae gradually broadened from base to apex (abruptly widened at base in *L.fengileana*; Fig. 2H); and apices of parameres rounded and constricted, lateral margins nearly straight (distinctly concave in posterior in *L.fengileana*).

##### Description.

Holotype (Fig. 2A–G). Body length 7.7 mm, width 3.0 mm. Body black; antennae, maxillary palpi, and legs blackish brown; dorsal surface shiny, with sparse punctures bearing short setae.

Head hexagonal, surface smooth, with dense, large punctation bearing short setae. Genae distinctly raised, surface without punctures in apical part, and sides with small punctures. Eyes ovate and prominent. Epistome trapezoidal, with anterior margin weakly emarginated; surface slightly convex at middle, with shallow, small punctures, and each lateral side with a longer seta near anterior angle. Fronto-clypeal suture indistinct, not depressed. Frons distinctive longitudinal convex at middle, with large, sparse large punctures; lateral parts depressed, with large, dense punctures. Vertex weakly convex, with large, sparse punctures on middle. Antennae slender, reaching pronotal base when directed backwards; antennomere III approximately 2.1× as long as antennomere II, the relative ratio of the length of antennomeres II–XI as follows: 0.16:0.33:0.26:0.24:0.26:0.26:0.26:0.26:0.28:0.42.

Pronotum widest at anterior 1/3, widened anteriorly and significantly convergent from anterior 1/3 to anterior margin; anterior margin slightly emarginated at middle; lateral margins neither marked nor beaded; basal margin neither bent downwards nor beaded; disc strongly convex, surface with large, sparse punctures, and distance between punctures 0.5–3.0× puncture diameter; anterior and posterior angles rounded, not produced. Prothoracic hypomera with punctures as large as those on disc, but with shorter setae. Prosternal process widest at middle and bent downwards behind coxae; surface with dense and large punctures bearing very short setae.

Elytra (Fig. 2C) elongate-oval, widest at middle, approximately 1.6× longer than wide; lateral sides arcuate; humeral angles rounded. Elytral surface smooth, with rows of punctures without striate, bearing very short setae; punctures in rows as large as those on pronotal disc; elytral intervals with few punctures nearly invisible, interval IX with three setigerous pores (one on anterior part, two on posterior part). Elytral apices significantly prolonged and with apex obtuse.

Abdomen ovoid, approximately 1.7× as long as wide. Surface convex, smooth, with punctures gradually became smaller from ventrites I–IV, bearing short setae; posterior part of ventrites IV distinctly convex transversely at posterior part before posterior margin; ventrites V with setae at posterior part longer than those on anterior part.

Legs (Fig. 2D–F) long and slender. Femora with sharp teeth near apex on inner sides; tooth on profemora rounded at apex, and meso- and metafemoral teeth acute and pointed at apex. All tibiae slender and distinctly hooked at inner apex; protibiae gradually becoming broader from base to apex, metatibiae slightly S-shaped on inner sides.

Aedeagus (Fig. 2G) subfusiform, length 2.2 mm, width 1.8 mm. Parameres trapezoidal, widest at base and narrowing to apex, with rounded apex; lateral sides of parameres shortly constricted before apex.

##### Sexual dimorphism.

Female. Body length 7.1 mm, width 3.0 mm. Apex of tibiae not hooked at inner sides.

##### Distribution.

China: Gansu.

##### Etymology.

The name of this species honors the late Prof. Jinchu Hu (China West Normal University, Nanchong City, China) who is a famous expert on the Giant Panda.

##### Note.

The specimens were collected by sifting leaf litter in a mixed forest. The paratype was preserved in 95% alcohol, and a hind leg was used to extract the whole genome. The mitochondrial gene COI of this new species is provided in Table 1.

#### ﻿ Laena bifoveolata

Taxon classificationAnimaliaColeopteraTenebrionidae

Reitter, 1889

C0145877-44A8-58A7-ADF6-3706D70680D2

Fig. 3A

Laena bifoveolata Reitter, 1889: 709; Schawaller 2001: 7; Schawaller 2008: 404; Wei et al. 2020: 523.

##### Examined specimens.

China – Ningxia Hui Autonomous Region • 4♂4♀;·Liupanshan, Longtan forestry station; 35.3898°N, 106.3451°E; elev. 1936 m, 2008-VI-23, Qiaohe Lou leg., IZAS – Gansu Province • 1♂ (in 95% ethanol); Dingxi City, Zhang County, Xinsizhen, Dishuiya; 34.6025°N, 104.5713°E; elev. 1930 m; 2022-VI-30; Qi Liu leg.; CWNU • 2♀ (in 95% ethanol); Hui County, Xiaolongshan, 33.6522°N, 106.2938°E; elev. 1920 m; 2022-VII-9; Qi Liu leg.; CWNU • 2♀ (in 95% ethanol); Qingshui County, Shanmenzhen, Dajicun; 34.2153°N, 106.3372°E; elev. 1784 m; 2022-VI-23; Qi Liu leg.; CWNU • 1♂4♀ (in 95% ethanol); Dingxi City, Zhang County, Malizhen, Huihuiliang; 34.5019°N, 104.7097°E; elev. 2300 m; 2022-VII-7; Qi Liu leg.; CWNU • 1♀ (in 95% ethanol); Qingshui County, Shanmen forest farm; 34.4056°N, 106.2222°E; elev. 1666 m; 2022-VI-21; Qi Liu leg.; CWNU.

**Figure 3.**
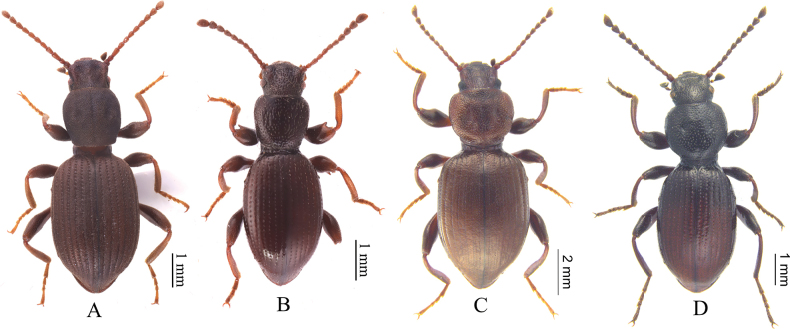
The four *Laena* species previously known from Gansu Province **A***L.bifoveolata***B***L.fengileana***C***Laenahaigouica***D***L.langmusica*.

##### Distribution.

China: Shaanxi, Ningxia, Gansu, Hubei, and Sichuan.

#### ﻿ Laena fengileana

Taxon classificationAnimaliaColeopteraTenebrionidae

Masumoto, 1996

93727F0A-5708-5F89-93B1-7804C7153368

Figs 2H , 3B

Laena fengileana Masumoto, 1996: 180; Schawaller 2001: 15; Schawaller 2008: 404; Yuan and Ren 2018: 698; Wei and Ren 2023: 79.

##### Examined specimens.

China – Gansu Province • 1♂ (in 95% ethanol); Li County, Shangpingxiang, Changankan; 34.1416°N, 104.8240°E; elev. 2550 m; 2022-VII-2; Qi Liu leg.; CWNU • 3♂ (in 95% ethanol); Li County, Taopingxiang, Nanshan; 34.0745°N, 104.8977°E; elev. 2190 m; 2022-VII-3; Qi Liu leg.; CWNU • 1♂1♀; Woniushan forest park; 34.4832°N, 104.8311°E; elev. 2650 m; 2022-VI-28; Qi Liu leg.; CWNU – Shaanxi Province • 2♂; Qinling Shan Mt. range, W pass on road Xi’an to Shagoujie, 45 km, SW Xi’an; 33°52′N, 108°46′E ; elev. 2800 m; 2001-VII-25; A. Metana leg.; MHBU.

##### Distribution.

China: Sichuan, Shaanxi, and Gansu.

#### ﻿ Laena haigouica

Taxon classificationAnimaliaColeopteraTenebrionidae

Schawaller, 2001

EC8CAFDE-253D-5862-9666-030751560347

Fig. 3C

Laena haigouica Schawaller, 2001: 19; Schawaller 2008: 405; Wei et al. 2020: 526; wei and Ren 2023: 79.

##### Examined specimens.

China – Sichuan Province • 1♂ (in 95% ethanol); Songpan, Huanglongxiang, Dawancun; elev. 2920 m; 2022-VII-23; Zhonghua Wei leg.; CWNU.

##### Distribution.

China: Gansu and Sichuan.

#### ﻿ Laena langmusica

Taxon classificationAnimaliaColeopteraTenebrionidae

Schawaller, 2001

92C1E974-3677-583B-BA37-8F71346FBE70

Fig. 3D

Laena langmusica Schawaller, 2001: 25; Schawaller 2008: 405; Yuan and Ren 2018: 699; Wei et al. 2020: 526.

##### Examined specimens.

China – Sichuan Province • 1♂1♀; West of Zhier (= Zier); elev. 4241 m; 28°20.87′N, 101°28.36′E; 5-VI-2004; R. Sehnai and M. Tryzna leg.; MHBU.

##### Distribution.

China: Shaanxi, Gansu, and Sichuan.

## ﻿Acknowledgments

We thank Mr. Qi Liu (Hebei University, Baoding, Hebei, China) for collecting the specimens, and Dr. Menglin Wang (China West Normal University, Nanchong, Sichuan, China) for revising the manuscript. We cordially thank reviewers Dr. Wolfgang Schawaller and Dr. Maxim Nabozhenko for their valuable comments on the manuscript.

## Supplementary Material

XML Treatment for Laena hui

XML Treatment for Laena bifoveolata

XML Treatment for Laena fengileana

XML Treatment for Laena haigouica

XML Treatment for Laena langmusica

## ﻿Additional information

### Author ORCIDs

Zhonghua Wei https://orcid.org/0000-0001-7349-9939

Guodong Ren https://orcid.org/0000-0001-5808-9122

### Data availability

The new sequences of the mitochondrial gene COI are available in NCBI (OR682144–OR682149).
